# Structural Genomic Analysis of SARS-CoV-2 and Other Coronaviruses

**DOI:** 10.3389/fgene.2022.801902

**Published:** 2022-04-08

**Authors:** Qiong Zhang, Huai-Lan Guo, Jing Wang, Yao Zhang, Ping-Ji Deng, Fei-Feng Li

**Affiliations:** ^1^ School of Pharmaceutical Sciences, Hubei University of Medicine, Shiyan, China; ^2^ Hubei Key Laboratory of Wudang Local Chinese Medicine Research, Hubei University of Medicine, Shiyan, China; ^3^ Hubei Biomedical Detection Sharing Platform in Water Source Area of South to North Water Diversion Project, Hubei University of Medicine, Shiyan, China; ^4^ School of Public Health, Hubei University of Medicine, Shiyan, China

**Keywords:** severe acute respiratory syndrome coronavirus 2 (SARS-CoV-2), common coronaviruses (C-CoVs), structural gene, evolution, intermediate hosts

## Abstract

Severe acute respiratory syndrome coronavirus-2 (SARS-CoV-2) is the causative agent of the coronavirus disease 2019 (COVID-19) pandemic. In this study, we conducted a comparative analysis of the structural genes of SARS-CoV-2 and other CoVs. We found that the sequence of the E gene was the most evolutionarily conserved across 200 SARS-CoV-2 isolates. The E gene and M gene sequences of SARS-CoV-2 and NC014470 CoV were closely related and fell within the same branch of a phylogenetic tree. The absolute diversity of E gene and M gene sequences of SARS-CoV-2 isolates was similar to that of common CoVs (C-CoVs) infecting other organisms. The absolute diversity of the M gene sequence of the KJ481931 CoV that can infect humans was similar to that of SARS-CoV-2 and C-CoVs infecting other organisms. The M gene sequence of KJ481931 CoV (infecting humans), SARS-CoV-2 and NC014470 CoV (infecting other organisms) were closely related, falling within the same branch of a phylogenetic tree. Patterns of variation and evolutionary characteristics of the N gene and S gene were very similar. These data may be of value for understanding the origins and intermediate hosts of SARS-CoV-2.

## Introduction

The coronaviruses (CoVs) are a large family of viruses that infect many organisms, including humans (Ma et al., 2020). The primary symptoms resulting from CoV infection are respiratory diseases and severe acute respiratory syndrome (Ashour et al., 2020). CoVs are enveloped viruses with a positive sense single stranded RNA genome. CoVs were first discovered in patients with the common cold in 1966 (Tyrrell and Bynoe 1966; Velavan and Meyer 2020).

Severe acute respiratory syndrome coronavirus-2 (SARS-CoV-2) belongs to the *Betacoronavirus* genus and the *Sarbecovirus* subgenus (Ceraolo and Giorgi 2020; Li F et al., 2020). Infection by SARS-CoV-2 results in a syndrome called coronavirus disease 2019 (COVID-19); the virus has caused a global pandemic, resulting in large numbers of illnesses and deaths [(An update on the epidemiological characteristics of novel coronavirus pneumoniaCOVID-19) 2020]. The main features of COVID-19 are high transmissibility and high mortality [Lai et al., 2020, (An update on the epidemiological characteristics of novel coronavirus pneumoniaCOVID-19) 2020]. Since the first patient with COVID-19 was identified (Lai, Shih, Ko, Tang and Hsueh 2020), more than 68 million additional cases have been confirmed globally with over 1.5 million deaths.

Many organisms have been considered as potential intermediate hosts of SARS-CoV-2 [Guo et al., 2020; Jiang and Shi 2020, (An update on the epidemiological characteristics of novel coronavirus pneumoniaCOVID-19) 2020; Zhang et al., 2020c; Zhou et al., 2020]. In a previous study, we concluded that SARS-CoV-2 may have evolved from a distant common ancestor of other common CoVs (C-CoVs), and may have persisted in an unidentified primary host for a long period (Li X et al., 2020). However, the origins and the intermediate hosts of SARS-CoV-2 remain unclear.

The SARS-CoV-2 genome is about 30 kb in size, making it one of the largest known viral RNA genomes. The genome contains four structural genes: S, E, M and N (Comas-Garcia 2019; Khailany et al., 2020). The “crown-like” appearance of SARS-CoV-2 results from the presence of the spike (S) glycoprotein (encoded by the S gene) on the surface of the virus (Jacofsky et al., 2020). The S protein binds to angiotensin-converting enzyme-2 (ACE2) and mediates fusion of the viral envelope with host cells (Lu et al., 2020). The other major SARS-CoV-2 envelope protein is the transmembrane (M) glycoprotein (encoded by the M gene) (Jacofsky et al., 2020). The main functions of the M protein are viral envelope formation and virion assembly (Ujike and Taguchi 2015; Jacofsky et al., 2020). The SARS-CoV-2 capsid and genomic RNA are linked by the basic (N) phosphoprotein (encoded by the N gene) (Khailany et al., 2020; Mousavizadeh and Ghasemi 2020). The other structural protein is the envelope (E) protein (encoded by the E gene), which is involved in virion assembly, release, and viral pathogenesis (Schoeman and Fielding 2019). The sequences of SARS-CoV-2 structural genes or proteins may contain information on the origins and intermediate hosts of the virus, which may be useful for vaccine development.

In this study, we analyzed the sequences of the structural genes of SARS-CoV-2 and C-CoVs that infect humans and other organisms. We aimed to understand variation and evolutionary characteristics of SARS-CoV-2 structural gene sequences.

## Materials and Methods

### Materials

We obtained structural gene sequences from 200 SARS-CoV-2 isolates, 126 C-CoVs that infect humans, and 53 C-CoVs that infect other organisms from the NCBI database (https://www.ncbi.nlm.nih.gov/sars-cov-2/).

### Analysis of Variation in SARS-CoV-2 Structural Gene Sequences

To analyze variation in the structural gene sequences of 200 SARS-CoV-2 isolates, we carried out multiple sequence alignments using Vector NTI software (Li et al., 2016). We analyzed the influence of mutations in structural gene sequences on the functions of structural proteins using DNAMAN software. We used MEGA-X software (Gorbalenya et al., 2020) to analyze the evolutionary features of SARS-CoV-2 structural gene sequences.

### Comparative Analysis of Structural Genes in SARS-CoV-2 and Other CoVs

We chose SARS-CoV-2 structural genes that showed sequence variation or evolutionary relatedness to C-CoVs for further analysis (Table 1). Using Vector NTI software and MEGA-X software (Kumar et al., 2018), we conducted a comparative sequence analysis of the structural gene sequences of SARS-CoV-2, C-CoVs that infect humans, and C-CoVs that infect other organisms.

**TABLE 1:** Analysis of structural gene sequences of 200 severe acute respiratory syndrome coronavirus-2 (SARS-CoV-2) isolates.

Genes	Size (nt)	Variations^1^	Variance rate^2^ (%)	Gene size variance rate^3^	SNPs	Mutations	For further analysis
E gene	228	2	1	0.44/10,000	MT263389, MT259248	—	MT263389, MT259248, ***MT263410*** ^6^
M gene	669	9	4.5	0.67/10,000	MT259252, MT263384, MT263410, MT263389, MT263443, MT263388, MT263422, MT263447	MT263397	MT263410, MT263389, MT263397, ***MT263074*** ^6^
N gene	908	28	14	1.54/10,000	MT263398, ***MT256917*** ^4^, ***MT256918*** ^4^, MT259270, MT263430, MT259267, MT263421, MT263451, MT258382, MT263435, MT263458, MT263395, MT259237	MT259237, MT259269, MT259274, MT263429, ***MT256917*** ^4^, ***MT256918*** ^4^, MT258379, MT259250, MT259263, MT263402, MT263074, MT263386, MT263410, MT263411, MT256924, MT263422, LC534419	MT263410, MT263074, MT263422, MT259237, MT259269, MT256917, MT263386, MT263411, MT258382, MT263398, MT259274, MT259270, MT263429, MT259267, MT263421, MT256924, LC534419, MT263435, MT263395, ***MT263389*** ^6^
S gene	3,822	89	44.5	1.16/10,000	MT259262, MT263410, MT259257, MT263441, MT263469, MT263386, MT259287, MT263074, MT259269, MT259227	MT263414, MT263460, MT263384, MT259249, ***MT263466(2)*** ^5^, MT259236, MT259276, MT263403, MT263412, MT263418, MT259262, MT259282, MT259253, MT262915, MT263457, MT263443, MT263393, MT263420, MT263385, MT263387, MT251973, MT251976, MT251979, MT258378, MT258379, MT258380, MT258382, MT258383, MT259235, MT259239, MT259240, MT259243, MT259244, MT259246, MT259248, MT259249, MT259250, MT259251, MT259256, MT259258, MT259260, MT259261, MT259263, MT259264, MT259265, MT259273, MT259277, MT263431, MT263436, MT259278, MT259281, MT259286, MT263074, MT263390, MT263391, MT263392, MT263394, MT263402, MT263406, MT263408, MT263411, MT263413, MT263415, MT263417, MT263426, MT263428, MT263432, MT263433, MT263437, MT263438, MT263439, MT263442, MT263445, MT263446, MT263459, MT263465, MT263467, MT263468	MT263410, MT263074-3, MT263466, MT263384, MT263443, MT259269, MT263386, MT259249, MT263414, MT259262, MT259257, MT259236, MT259282, MT263441, MT262915, MT259287, MT251973, MT263393, MT263385, MT259253, MT263457, MT263420, MT259227, ***MT263389*** ^6^

Notes: ^1^Variations include single nucleotide polymorphisms (SNPs) and mutations.

2 Variance rate= (variations/200) × 100%.

3 Gene size variance rate= (variations/200/gene size) × 10,000/10,000.

4 There were two variations in the MT256917 and MT256918 CoVs, respectively.

5 There were two mutations in the MT263466 CoV.

6 No variation controls for further analysis of structural genes.

## Results

### Genomic Analysis of SARS-CoV-2 Structural Gene Sequences

**FIGURE 1:**
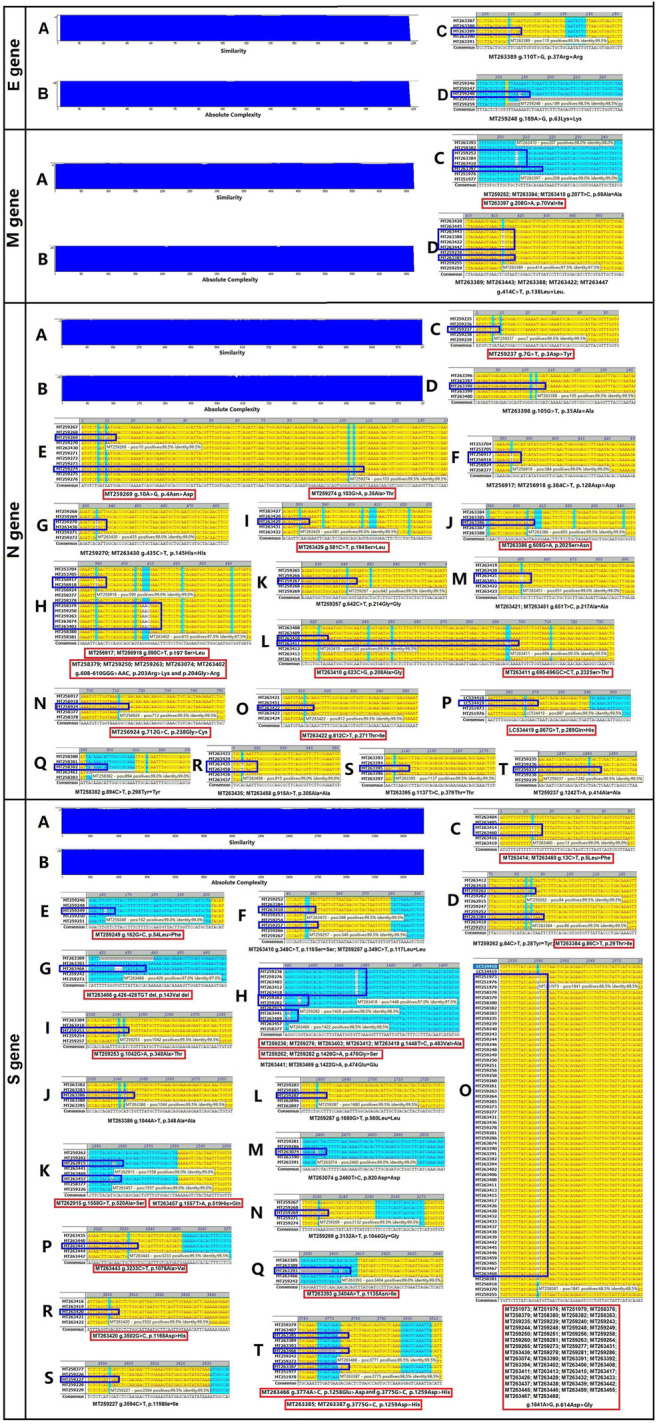
Absolute diversity and variations in the structural genes of 200 severe acute respiratory syndrome coronavirus-2 (SARS-CoV-2) isolates. The similarity and absolute diversity in structural genes sequences were very high. Two SARS-CoV-2 isolates had two single nucleotide polymorphisms (SNPs) within the E gene, nine isolates had three variations (one mutation and two SNPs) within the M gene, 28 strains had 22 variations (13 mutations and nine SNPs) within the N gene, and 89 strains had 25 variations (16 mutations and nine SNPs) within the S gene.

The four structural genes encoded in the SARS-CoV-2 genome are E (228 nt), M (669 nt), N (908 nt), and S (3,822 nt). As shown in Figure 1, the similarities and absolute diversities of SARS-CoV-2 structural gene sequences were very high (Figure 1 A,B).

Two SARS-CoV-2 isolates had two single nucleotide polymorphisms (SNPs) within the E gene (Figure 1 C,D and Table 1), nine isolates had three variations (one mutation and two SNPs) within the M gene (Figure 1 C,D and Table 1), 28 isolates had 22 variations (13 mutations and nine SNPs) within the N gene (Figure 1, C–T and Table 1) and 89 isolates had 25 variations (16 mutations and nine SNPs) within the S gene (Figure 1, C–T and Table 1).

The variance rates (VRs) of structural genes among the 200 SARS-CoV-2 isolates were 1% (E), 4.5% (M), 14% (N) and 44.5% (S) (Table 1). The gene size variance rates (GSVRs) of the four genes were 0.44/10,000 (E), 0.67/10,000 (M), 1.54/10,000 (N) and 1.16/10,000 (S) (Table 1). The sequence of the E gene was the most highly conserved across the 200 SARS-CoV-2 isolates.

### Influence of Mutations in SARS-CoV-2 Structural Genes on the Features of Structural Proteins

We identified 30 mutations within the structural genes of 200 SARS-CoV-2 isolates. Subsequently, we analyzed the influence of these mutations on the features of structural proteins. As shown in Supplementary Figure S1, the Val70→Ile substitution in the M gene of the MT263397 isolate had little effect on the transmembrane segment of the M protein.

In the N gene, six mutations affected N protein hydrophobicity, three mutations affected protein hydrophilicity, 10 mutations affected protein secondary structure, and four mutations affected the transmembrane segment (Supplementary Figure S2).

One mutation in the S gene affected S protein hydrophobicity, one mutation affected protein hydrophilicity, and three mutations affected protein secondary structure (Supplementary Figure S3).

In general, mutations in the N gene of SARS-CoV-2 isolates occurred between amino acid residues 200 to 300 and had large impacts on the function of the protein (Figure 1 and Supplementary Figure S2).

### Phylogenetic Analysis of SARS-CoV-2 Structural Gene Sequences

**FIGURE 2:**
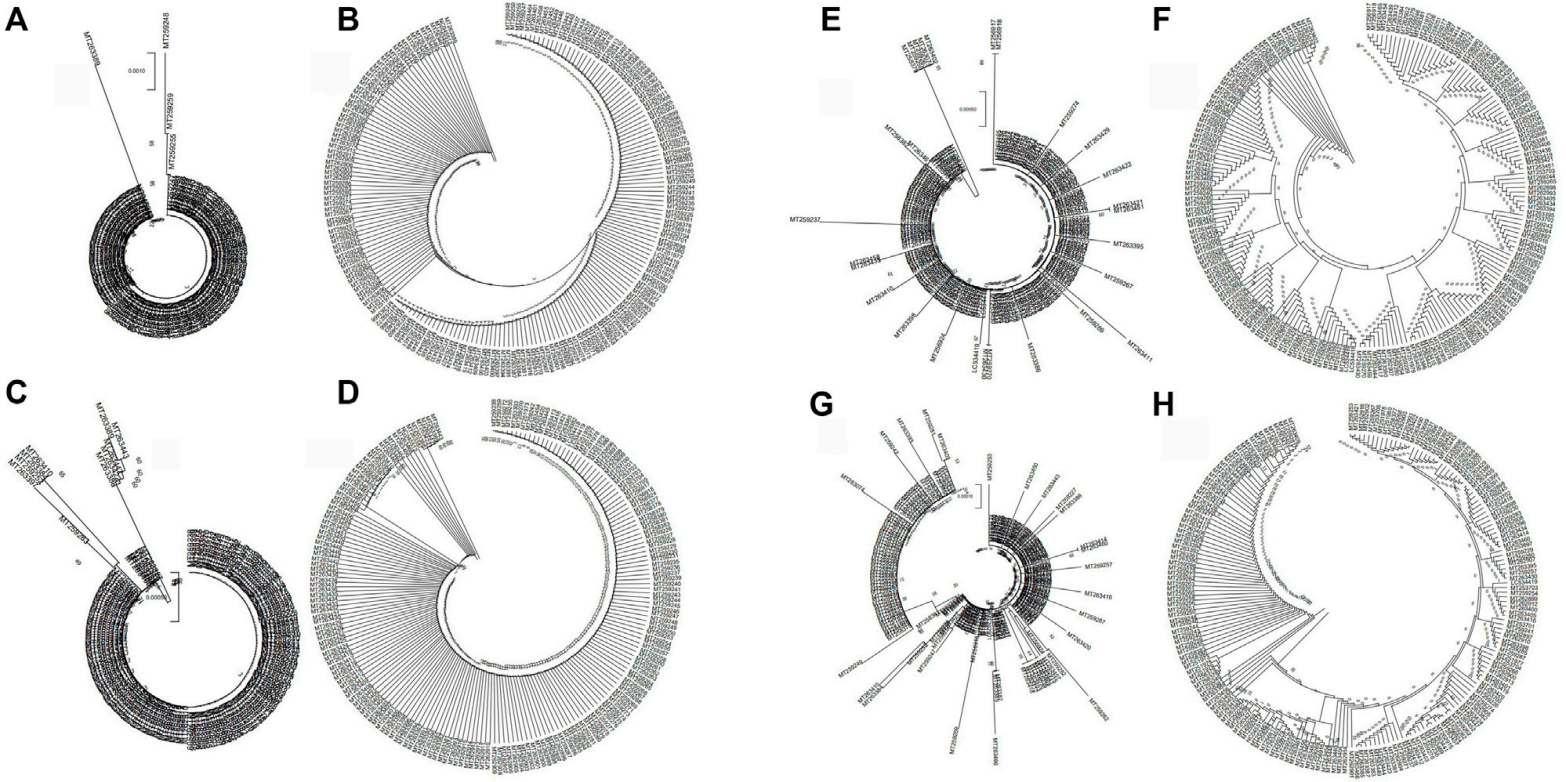
Evolutionary characteristics of the structural genes of 200 severe acute respiratory syndrome coronavirus-2 (SARS-CoV-2) isolates. **(A,B)**: The two isolates with single nucleotide polymorphisms (SNPs) in the E gene were evolutionary distinct. **(C,D)**: The nine isolates with variations in the M gene were evolutionary distinct. **(E,F)**: The 28 isolates with variations in the N gene were evolutionarily distinct. **(G,H)**: The 88 strains with variations in the S gene evolutionarily distinct.

Next, we analyzed the evolutionary characteristics of the structural genes of SARS-CoV-2 isolates. As shown in Figure 2, the SARS-CoV-2 structural genes showing increased variation also showed distinct evolutionary features. The sequence of the E gene was the most evolutionarily conserved across the 200 SARS-CoV-2 isolates (Figure 2). We selected the sequences of structural genes that showed variation and evolutionary relatedness with C-CoVs for further analysis (Table 1).

### Comparative Analysis of Structural Gene Sequences of SARS-CoV-2 and C-CoVs That Infect Humans

**FIGURE 3:**
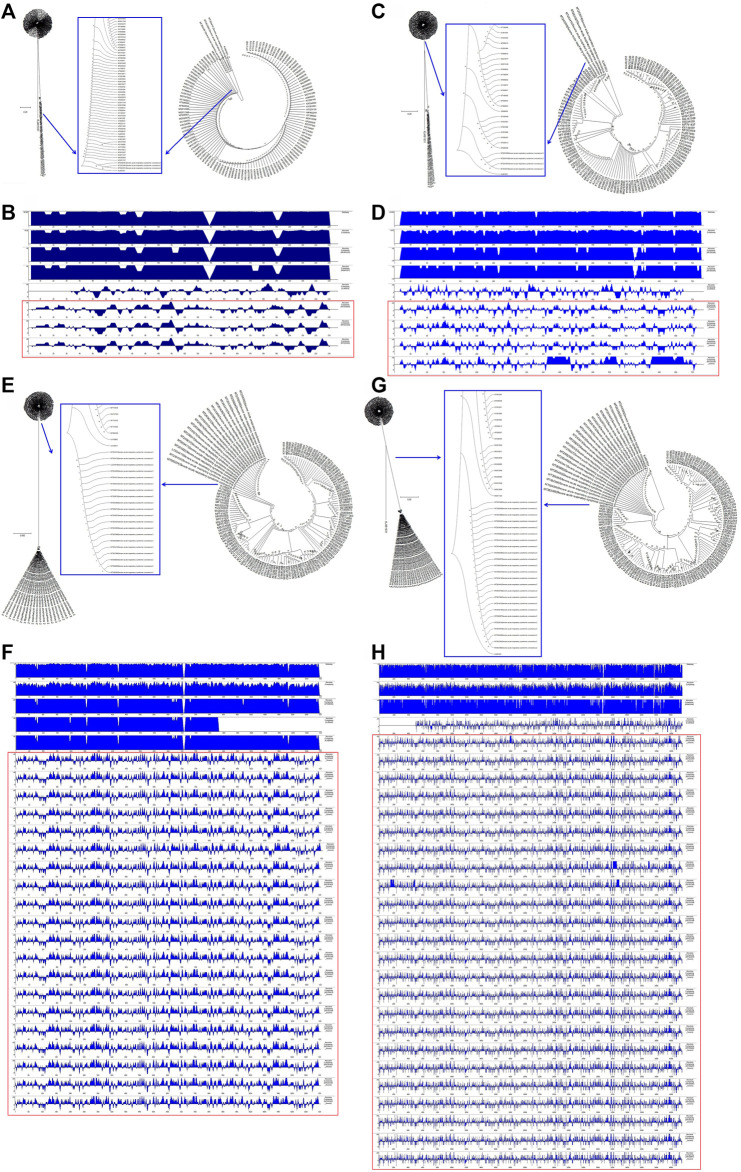
Evolutionary characteristics and absolute diversity of structural genes in severe acute respiratory syndrome coronavirus-2 (SARS-CoV-2) isolates and common coronaviruses (C-CoVs) that infect humans. **(A)**: The E gene sequences of SARS-CoV-2 isolates were evolutionary intermediates between KJ481931 and MG011357. **(C)**: The M gene sequences of SARS-CoV-2 isolates were evolutionary intermediates between KJ48193 and a group of C-CoVs (KP209309, KY581691, KY581689, KY581686, KP209307, KP209313, and KP209306). **(B,D)**: The absolute diversities of the E and M gene sequences within the KJ481931 C-CoV were similar to those of the E and M gene sequences of SARS-CoV-2 isolates. **(E,G)**: The N and S gene sequences of SARS-CoV-2 isolates were evolutionarily distinct. **(F,H)**: The absolute diversities of the N and S gene sequences of SARS-CoV-2 isolates differed from those of all C-CoVs that infect humans.

To understand the relationships between the structural genes of SARS-CoV-2 and C-CoVs that also infect humans, we carried out a comparative sequence analysis of selected structural gene sequences from SARS-CoV-2 (Table 1) and C-CoVs that infect humans. As shown in Figure 3, the E gene sequences of SARS-CoV-2 isolates were evolutionary intermediates between KJ481931 and MG011357 (Figure 3A). In terms of their E gene sequences, SARS-CoV-2 and KJ481931 were the most closely related evolutionarily (Figure 3A), and the absolute diversities of the E gene sequences of these two CoVs was similar (Figure 3B).

The M gene sequences of SARS-CoV-2 isolates were evolutionary intermediates between KJ48193 and a group of other CoVs (KP209309, KY581691, KY581689, KY581686, KP209307, KP209313, and KP209306). The M gene sequences of SARS-CoV-2 and KJ481931 were the most closely related evolutionarily (Figure 3C), and the absolute diversities of the M gene sequences of these two CoVs was similar (Figure 3D).

The N gene and S gene sequences of SARS-CoV-2 isolates were evolutionarily distinct (Figure 3E and Figure 3G). The absolute diversities of N gene sequences in SARS-CoV-2 isolates differed from those of all other C-CoVs (Figure 3F). However, the S gene sequences of SARS-CoV-2 isolates and KJ481931 were the most closely related evolutionarily (Figure 3G), and the absolute diversities of the S gene sequences of these two CoVs were similar (Figure 3H).

### Comparative Analysis of Structural Gene Sequences of SARS-CoV-2 and C-CoVs That Infect Other Organisms

**FIGURE 4:**
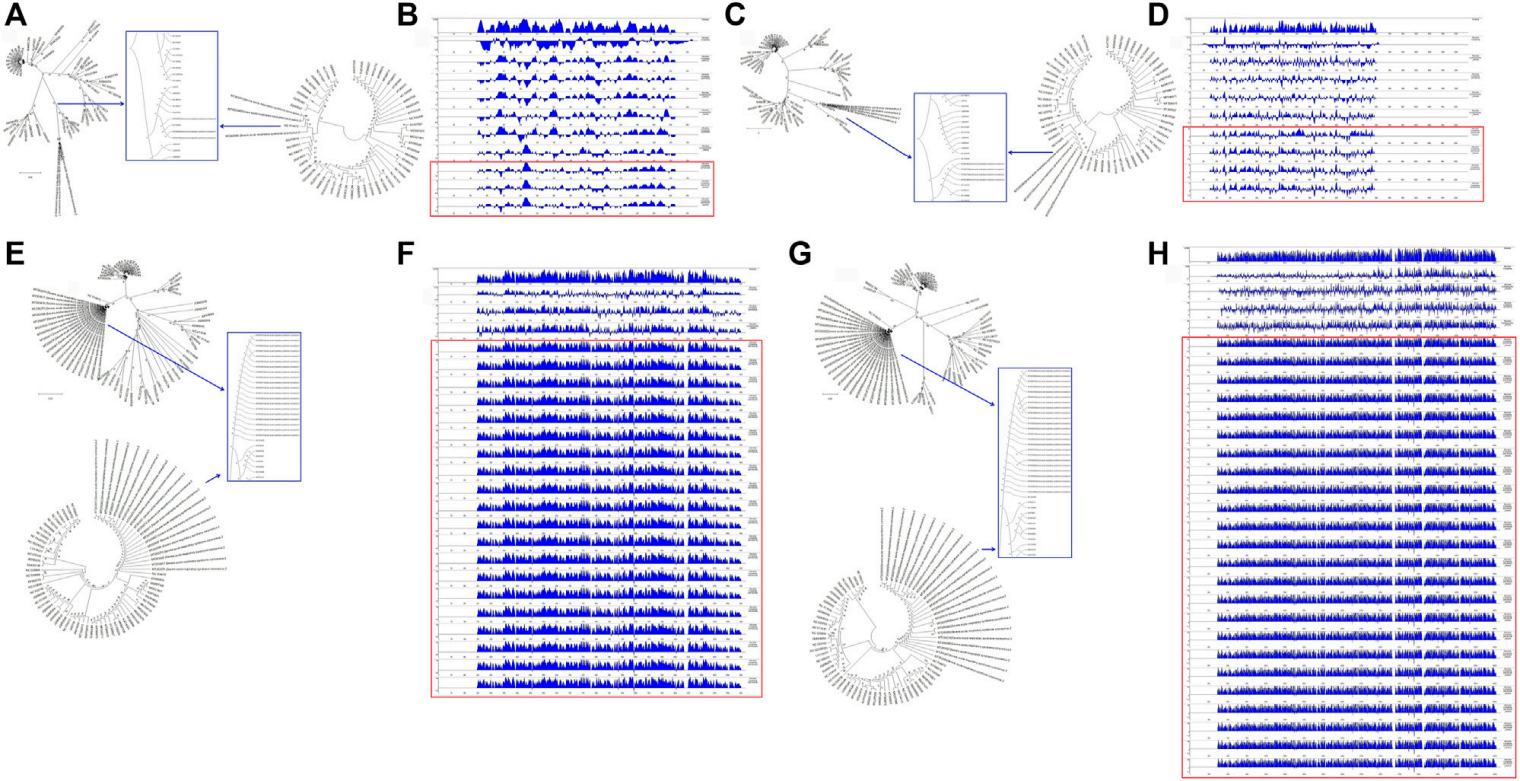
Evolutionary characteristics and absolute diversities of structural genes in severe acute respiratory syndrome coronavirus-2 (SARS-CoV-2) isolates and common coronaviruses (C-CoVs) that infect other organisms. **(A,C)**: The E and M gene sequences of SARS-CoV-2 isolates were evolutionarily intermediates between C-CoVs that infect other organisms. **(A)**: In terms of E gene sequences, SARS-CoV-2 isolates were most closely related to the C-CoVs NC014470, DQ415914, NC026011, NC006213, JN874559 and U00735. **(B)**: The absolute diversities of the E gene sequences of the C-CoVs NC014470, DQ415914, NC026011, NC006213, JN874559, and U00735 were similar to those of the E gene sequences of SARS-CoV-2 isolates. **(C)**: In terms of M gene sequences, SARS-CoV-2 isolates were most closely evolutionarily related to the C-CoVs NC014470, EF065513 and NC030886. **(D)**: The absolute diversities of the M gene sequences of the C-CoVs NC014470, EF065513 and NC030886 were similar to those of M gene sequences of SARS-CoV-2 isolates. **(E,G)**: In terms of N and S gene sequences, SARS-CoV-2 isolates were most closely evolutionarily related to the C-CoV NC014470, forming a separate clade. **(F)**: The absolute diversity of N gene sequences of SARS-CoV-2 isolates was similar to that of the C-CoV NC014470. **(H)**: The absolute diversity of the S gene sequence of the C-CoV NC014470 was similar to those of other C-CoVs.

To understand the relationships between the structural genes of SARS-CoV-2 and C-CoVs that infect other organisms, we carried out a comparative sequence analysis of selected structural gene sequences from SARS-CoV-2 (Table 1) and C-CoVs that infect other organisms. As shown in Figure 4, the E gene sequences of SARS-CoV-2 isolates were most closely evolutionarily related to NC014470, DQ415914, NC026011, NC006213, JN874559, and U00735l; NC014470 was also located within the same branch of a phylogenetic tree as SARS-CoV-2 isolates (Figure 4A). The absolute diversities of E gene sequences from NC014470, DQ415914, NC026011, NC006213, JN874559, and U00735 were similar to those of E gene sequences from SARS-CoV-2 isolates (Figure 4B).

The M gene sequences of SARS-CoV-2 isolates were most closely related to NC014470, EF065513 and NC030886 (Figure 4C). The absolute diversities of M gene sequences from NC014470, EF065513 and NC030886 were similar to those of M gene sequences from SARS-CoV-2 isolates (Figure 4D).

In terms of N gene and S gene sequences, SARS-CoV-2 was most closely evolutionarily related to NC014470; these two CoVs formed a separate clade in a phylogenetic tree (Figure 4E and Figure 4G). The absolute diversity of N gene sequences from SARS-CoV-2 isolates was similar to that of the N gene sequence of NC014470 (Figure 4F). However, the absolute diversity of the S gene sequence from NC014470 was more similar to those of the S gene sequences of other C-CoVs (Figure 4H).

### Comparative Analysis of Structural Gene Sequences of SARS-CoV-2 and C-CoVs That Infect Humans and Other Organisms

**FIGURE 5:**
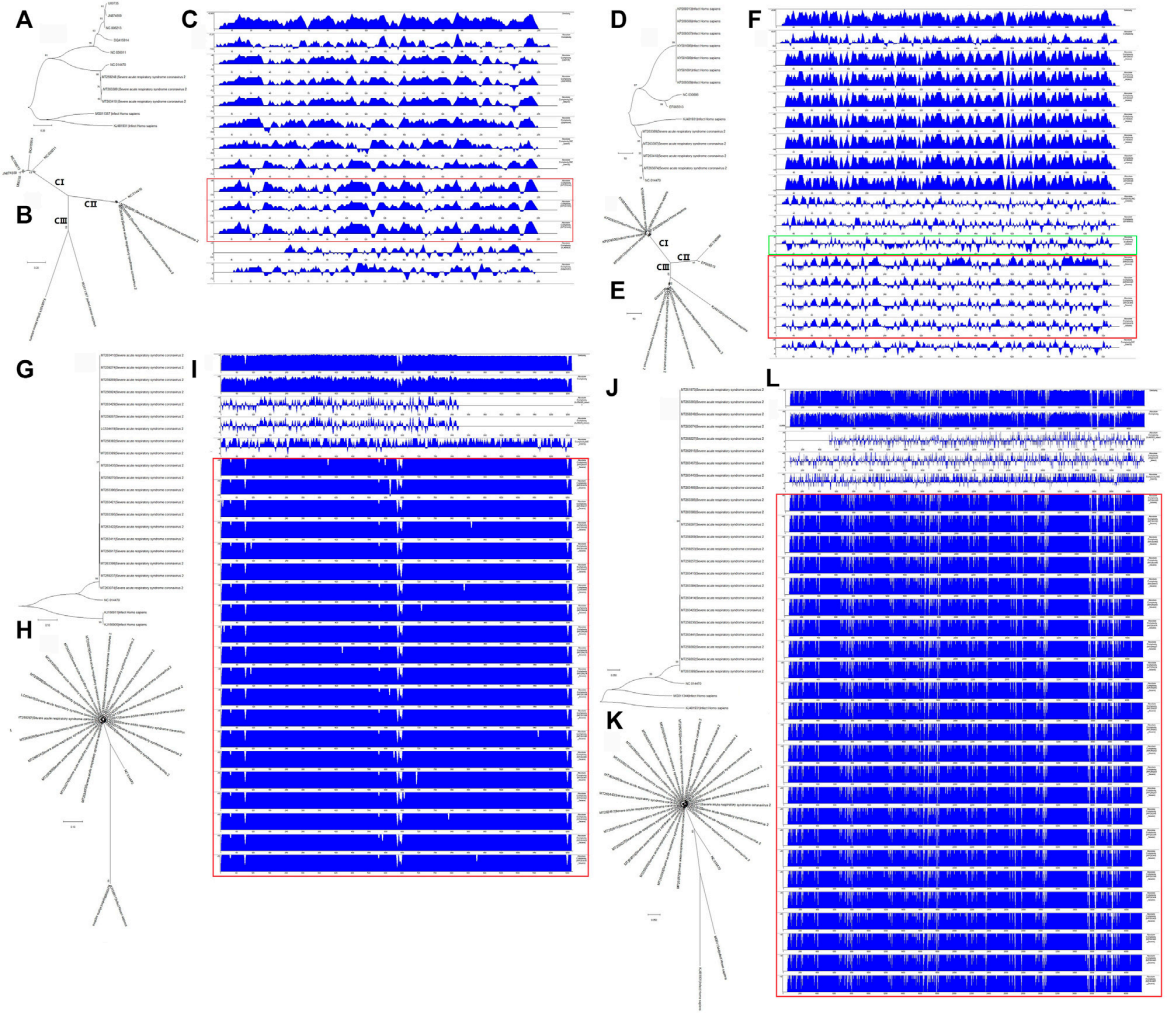
Evolutionary characteristics and absolute diversities of structural genes of severe acute respiratory syndrome coronavirus-2 (SARS-CoV-2) isolates and common coronaviruses (C-CoVs). **(A,B,D,E)**: The E and M gene sequences of SARS-CoV-2 isolates and common CoVs could be grouped into three clades (CI, CII and CIII). A, B: The E gene sequences of SARS-CoV-2 isolates and C-CoV NC014470 were evolutionary intermediates between C-CoVs that infect humans and other organisms. **(C)**: The absolute diversities of E gene sequences of SARS-CoV-2 isolates were more similar to those of C-CoVs that infect other organisms. **(D,E)**: The M gene sequences of SARS-CoV-2 isolates, NC014470 (infecting other organisms) and KJ481931 (infecting humans) were closely related and grouped together in the same branch of a phylogenetic tree. The M gene sequences of SARS-CoV-2 isolates were evolutionary intermediates between those of NC014470 (infecting other organisms) and KJ481931 (infecting humans). **(F)**: The absolute diversities of M gene sequences of SARS-CoV-2 isolates were more similar to those of C-CoVs infecting other organisms. **(F)** (Green box): The absolute diversity of M gene sequences of KJ481931 (infecting humans) was more similar to those of M gene sequences from SARS-CoV-2 isolates and C-CoVs that infect other organisms. **(G,H,J,K)**: The N and S gene sequences of SARS-CoV-2 strains grouped closely together on the same branch of an evolutionary tree. **(G,H,J,K)**: The N and S gene sequences of NC014470 were located between those of SARS-CoV-2 isolates and C-CoVs that infect humans. **(I,L)**: The absolute diversities of N and S gene sequences of SARS-CoV-2 isolates were unlike those of C-CoVs.

We next wanted to analyze the evolutionary relationships among the structural genes of SARS-CoV-2 and C-CoVs that infect humans and other organisms. We performed a comparative sequence analysis of the structural genes from SARS-CoV-2 isolates (Table 1) and those from C-CoVs (Table 2). As shown in Figure 5, the E gene sequences of SARS-CoV-2 isolates and C-CoVs could be grouped into three clades (CI, CII and CIII) (Figures 5A,B). In terms of their E gene sequences, SARS-CoV-2 isolates were most closely related to NC014470; these two CoVs represented evolutionary intermediates in the phylogenetic tree between C-CoVs that infect humans and those that infect other organisms (Figures 5A,B). The absolute diversity of E gene sequences of SARS-CoV-2 isolates was most similar to that of the E gene sequences of C-CoVs that infect other organisms (Figure 5C).

**TABLE 2:** Analysis of structural gene sequences of common coronaviruses (C-CoVs) evolutionarily related to severe acute respiratory syndrome coronavirus-2 (SARS-CoV-2).

Genes	C-CoVs infecting humans	C-CoVs other organisms
E gene	KJ481931, MG011357	NC014470, DQ415914, NC026011, NC006213, JN874559, U00735
M gene	KJ481931, KP209309, KY581691, KY581689, KY581686, KP209307, KP209313, KP209306	NC014470, EF065513, NC030886
N gene	KJ156911, KJ156905	NC014470
S gene	KJ481931, MG011344	NC014470

The M gene sequences of SARS-CoV-2 isolates and C-CoVs could be also grouped into three clades (CI, CII and CIII) (Figures 5D,E). The M gene sequences of SARS-CoV-2 isolates were evolutionary intermediates between NC014470 (infecting other organisms) and KJ481931 (infecting humans); SARS-CoV2 isolates grouped closely together in a same branch of the phylogenetic tree (Figures 5D,E). The absolute diversity of the M gene sequences of SARS-CoV-2 isolates was more similar to those of the M gene sequences of C-CoVs that infect other organisms (Figure 5F). However, the absolute diversity of the M gene sequence of KJ481931 (infecting humans) was more similar to that of M gene sequences from SARS-CoV-2 isolates and C-CoVs that infect other organisms (Figure 5F).

The N gene sequences of SARS-CoV-2 isolates were closely related and grouped together within the same branch of a phylogenetic tree (Figures 5G,H). The N gene sequence of NC014470 was an evolutionary intermediate between SARS-CoV-2 isolates and C-CoVs that infect humans (Figures 5G,H). The absolute diversity of the N gene sequences of SARS-CoV-2 isolates differed from the absolute diversity of the N gene sequences of C-CoVs (Figure 5I).

The evolutionary features and absolute diversities of the S gene sequences of SARS-CoV-2 isolates and C-CoVs that infect other organisms or humans were very similar to those of the N gene sequences (Figures 5J–L).

## Discussion

Genetic information determines the functions and characteristics of biological factors and organisms. Gene annotation and evolutionary analysis are important steps in interpreting sequence information (Khailany et al., 2020). In this work, we profiled variations in the structural gene sequences of SARS-CoV-2 isolates. We analyzed the evolutionary characteristics and absolute diversities of structural gene sequences of SARS-CoV-2 isolates and C-CoVs that infect humans and other organisms.

CoVs are positive-single-stranded RNA viruses. The major symptoms caused by CoV infection are respiratory tract infections. SARS-CoV, Middle East Respiratory Syndrome (MERS)-CoV and SARS-CoV-2 are three highly contagious and deadly CoVs that have caused outbreaks in humans (Singh Tomar and Arkin 2020). The genomes of SARS-CoV and SARS-CoV-2 share approximately 80% identity, but are distinct from those of other C-CoVs that infect humans (Lu et al., 2020, The species Severe acute respiratory syndrome-related coronavirus: classifying 2019-nCoV and naming it SARS-CoV-2 2020).

The SARS-CoV-2 genome including four structural genes encoding structural proteins: E, M, S and N (Comas-Garcia 2019). The functions of the E protein include assembly, release, and pathogenesis of CoVs (Schoeman and Fielding 2019). Important features of the E gene and protein are their small size and the high hydrophobicity of the E protein. Those features suggests that the E protein may act as a viroporin, and that CoVs lacking the E protein may be less virulent. The E protein many serve as a vaccine candidate (Fett et al., 2013; Regla-Nava et al., 2015). In this work, using the genome sequences of 200 SARS-CoV-2 isolates, we found that only two isolates had SNPs within the E gene. The sequence of the E gene was the most highly conserved across the 200 SARS-CoV-2 isolates.

The genomes of many CoVs contain an E gene, including SARS-CoV (Torres et al., 2006; Parthasarathy et al., 2008), MERS-CoV (Surya et al., 2015), human CoV 229E (Wilson et al., 2006), and SARS-CoV-2. In terms of their E gene sequences, we found the SARS-CoV-2 was most closely evolutionarily related to NC014470 [a C-CoV that infects bats (Drexler et al., 2010)]; these two CoVs were evolutionary intermediates between C-CoVs that infect humans and those that infect other organisms. The absolute diversity of the E gene sequences of SARS-CoV-2 isolates was more similar to that of E gene sequences from C-CoVs that infect other organisms.

The genetic and evolutionary features of M gene sequences within the 200 SARS-CoV-2 isolates were very similar to those of E gene sequences. As a major envelope protein, the M protein is responsible for viral envelope formation and virion assembly (Ujike and Taguchi 2015; Jacofsky et al., 2020). Here, we found that nine of 200 isolates showed variations (one mutation and eight SNPs) in the M gene. The VR and GSVR of the M gene were slightly higher than those of the E gene. However, the M protein is a major envelope protein (Ujike and Taguchi 2015), and the mutation (Val70→Ile) in the M gene of MT263397 had little impact on the transmembrane segment of the M protein. The M gene and protein is another good candidate for SARS-CoV-2 vaccine development.

The evolutionary features of M gene sequences were very interesting. The M gene sequences of SARS-CoV-2 isolates were evolutionary intermediate between those of NC014470 (infecting other organisms) and KJ481931 [infecting humans; (Marthaler et al., 2014)]; the M gene sequences of these CoVs were grouped closely together within the same branch of a phylogenetic tree. The absolute diversity of the M gene sequence from KJ481931 was more similar to that of M gene sequences from SARS-CoV-2 isolates and to those of M gene sequences of C-CoVs that infect other organisms.

During CoV infection, the N protein and viral RNA enter host cells together, where they are involved in viral assembly, release and genome replication (Narayanan et al., 2003). In the early stages of infection, antibodies against the N protein are highly specific (Shi et al., 2003; Leung et al., 2004; Tan et al., 2004). In this study, we found that 28 of 200 SARS-CoV-2 isolates showed a total of 22 variations within the N gene. Mutations mainly occurred between amino acid residues 200 to 300 and had a large impact on N protein function.

The genetic and evolutionary features of N and S structural genes within the 200 SARS-CoV-2 isolates were very similar. The VRs of N and S genes were 14 and 44.5%, respectively. However, the S gene sequence is longer than the N gene sequence (Khailany et al., 2020). The GSVR of the S gene was 1.16/10,000, lower than that of the N gene (1.54/10,000). We identified 58 isolates bearing the same variation (Asp614→Gly), but mutations in the S gene had little effect on protein function. The N gene sequence was less conserved than the S gene sequence.

The main function of the S protein is to mediate CoV entry into host cells (Tortorici and Veesler 2019). Among the four structural proteins, the S protein is the largest (Khailany et al., 2020). In the S protein, SARS-CoV-2 and SARS-CoV share 76% amino acid identity (de Groot 2006; Zhang et al., 2020a). Entry of SARS-CoV-2 into host cells can be prevented by antibodies raised against SARS-CoV (Hoffmann et al., 2020). The S protein of SARS-CoV-2 shared 93 and 97% amino acid identity with Bat CoV RaTG13 and Pangolin-CoV, respectively (Zhang et al., 2020b; Special Expert Group for Control of the Epidemic of Novel Coronavirus Pneumonia of the Chinese Preventive Medicine Association, 2020; Zhou et al., 2020). These results strongly suggest potential intermediate hosts based on conservation of the S protein. However, in our study we found that S gene sequences of SARS-CoV-2 isolates were evolutionarily independent in a phylogenetic tree, with a relatively large evolutionary distance separating the S genes of SARS-CoV-2 and C-CoVs. The absolute diversity of S gene sequences within SARS-CoV-2 isolates was also unlike those of S genes sequences from all the other C-CoVs.

## Conclusion

On the basis of these results, we conclude that the E and M structural genes of SARS-CoV-2 and the NC014470 and KJ481931 CoVs are important for understanding the origins and intermediate hosts of SARS-CoV-2.

## Data Availability

The original contributions presented in the study are included in the article/Supplementary Material, further inquiries can be directed to the corresponding authors.

## Supplementary Material

Supplementary Figure S1 Influence of a mutation in the M gene (g.208G→A, p.70Val→Ile) of the MT263397 coronavirus on protein structure and function. The mutation had little effect on the transmembrane segment of the M protein.

Supplementary Figure S2 Influence of mutations in the N genes of 200 severe acute respiratory syndrome coronavirus-2 (SARS-CoV-2) isolates on protein structure and function. Six mutations (MT259237, p.3Asp→Tyr; MT263429, p.194Ser→Leu; MT256917 and MT256918, p.197Ser→Leu; MT263410, p.208Ala→Gly; MT256924, p.238Gly→Cys; and MT263422, p.271Thr→Ile) had effects on protein hydrophobicity. Three mutations (MT259237, p.3Asp→Tyr; MT263429, p.194Ser→Leu; MT256917 and MT256918, p.197Ser→Leu) had effects on protein hydrophilicity. Ten mutations (MT259237, p.3Asp→Tyr; MT259274, p.35Ala→Thr; MT263429, p.194Ser→Leu; MT256917 and MT256918, p.197Ser>→Leu; MT263386, p.202Ser→Asn; MT258379, MT259250, MT259263, MT263074, and MT263402, p.203Arg→Lys and p.204Gly→Arg; MT263411, p.232Ser→Thr; MT256924, p.238Gly→Cys; MT263422, p.271Thr→Ile; and LC534419, p.289Gln→His) had effects on protein secondary structure. Four mutations (MT259237, p.3Asp→Tyr; MT263386, p.202Ser→Asn; MT258379, MT259250, MT259263, MT263074, and MT263402, p.203Arg→Lys and p.204Gly→Arg; and MT263422, p.271Thr→Ile) had effects on protein transmembrane segments.

Supplementary Figure S3 Influence of mutations in the S genes of 200 severe acute respiratory syndrome coronavirus-2 (SARS-CoV-2) isolates on protein structure and function. One mutation in the S gene affected protein hydrophobicity (MT263384, p.29Thr→Ile) and one mutation affected hydrophilicity (MT251973 and MT251976, p.614Asp→Gly). Three mutations (MT259253, p.348Ala→Thr; MT263466, p.1258Glu→Asp and p.1259Asp→His; MT263385, MT263387, p.1259Asp→His) in the S gene had effects on protein secondary structure.
