# Supplementary figures and images for: Structural Genomic Analysis of SARS-CoV-2 and Other Coronaviruses
Source: Front Genet. 2022 Apr 8;13:801902. doi: 10.3389/fgene.2022.801902 (PMC9024071; doi:10.3389/fgene.2022.801902)

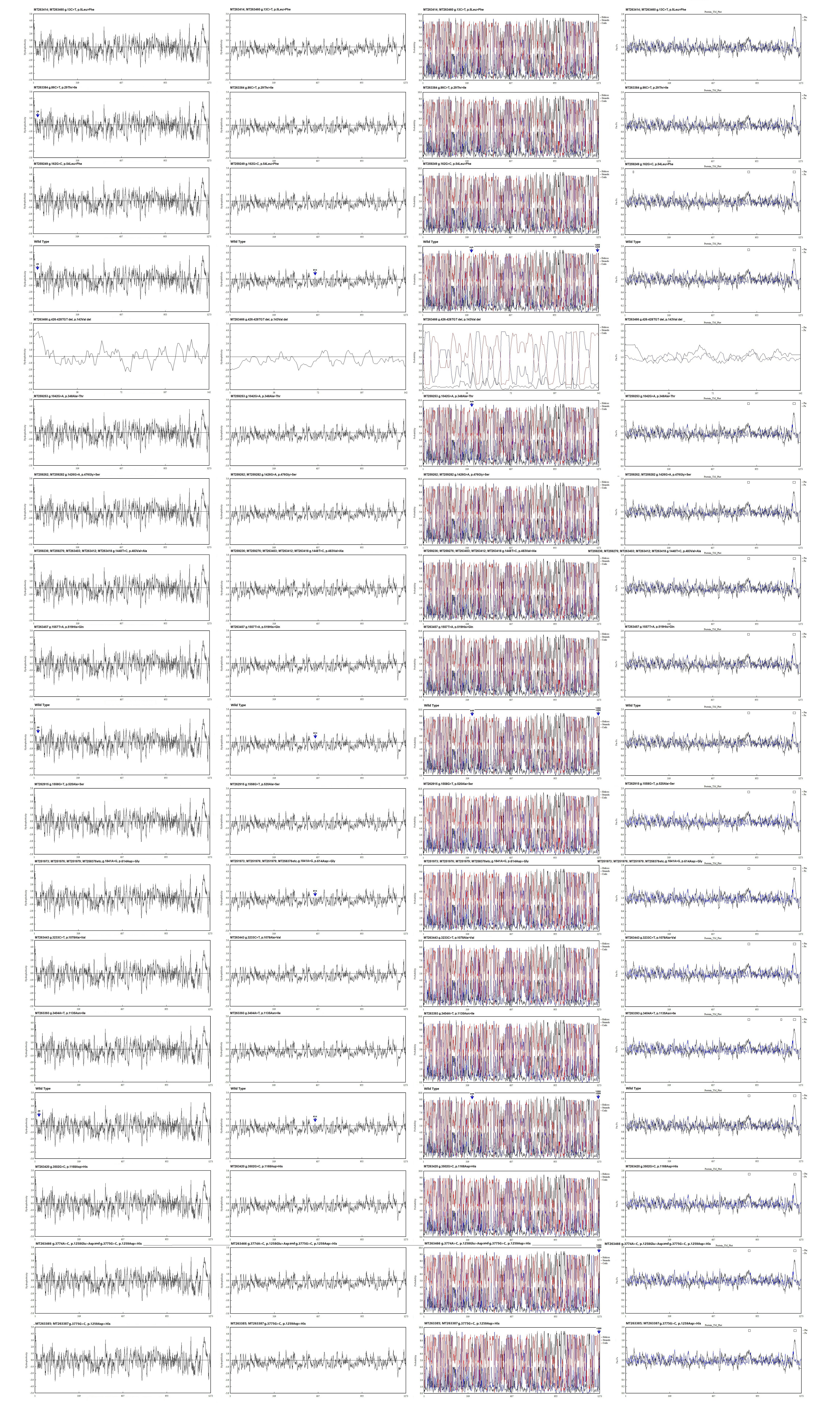

Supplement: Supplementary file 1 [file Image3.TIF]

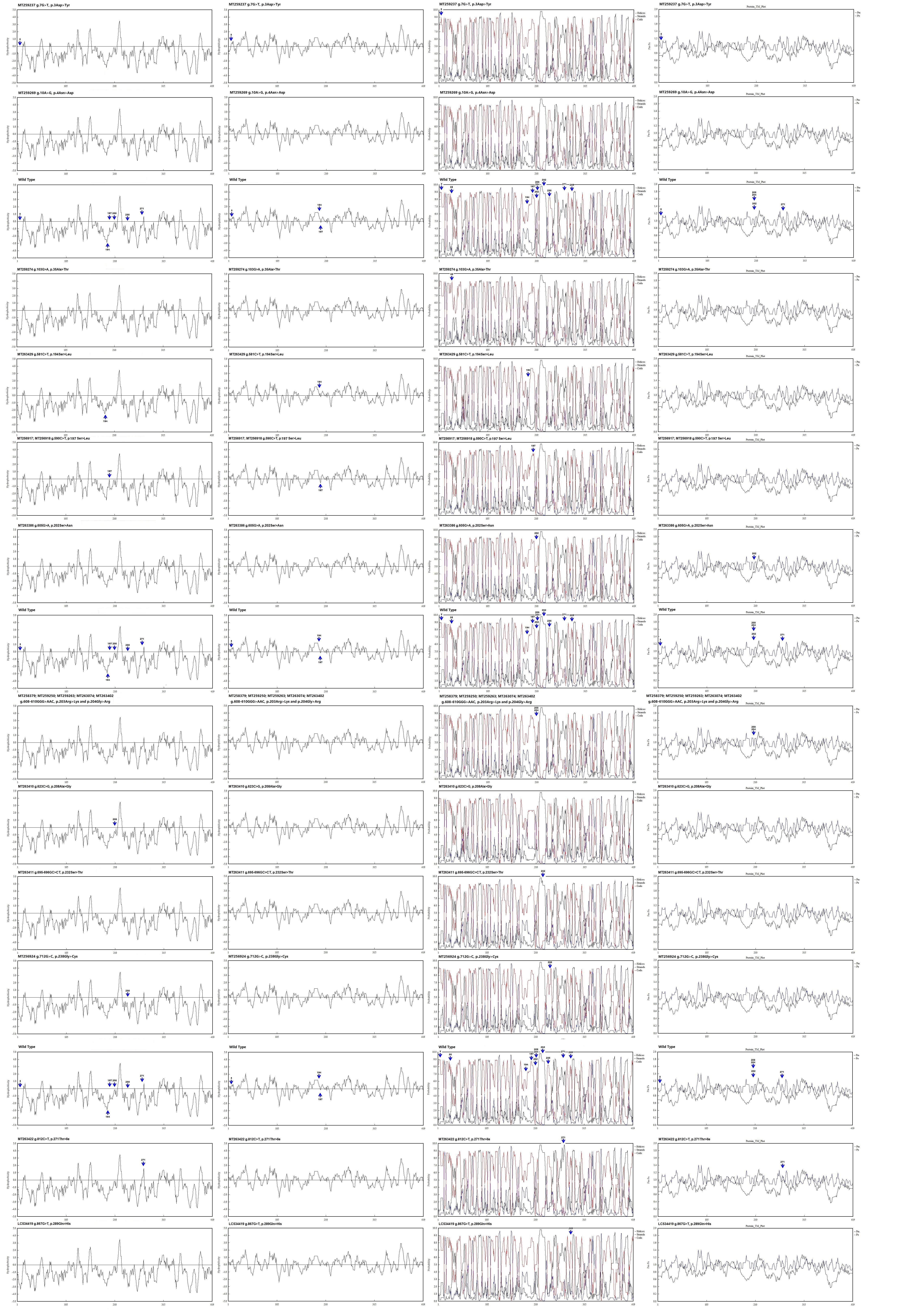

Supplement: Supplementary file 2 [file Image2.TIF]

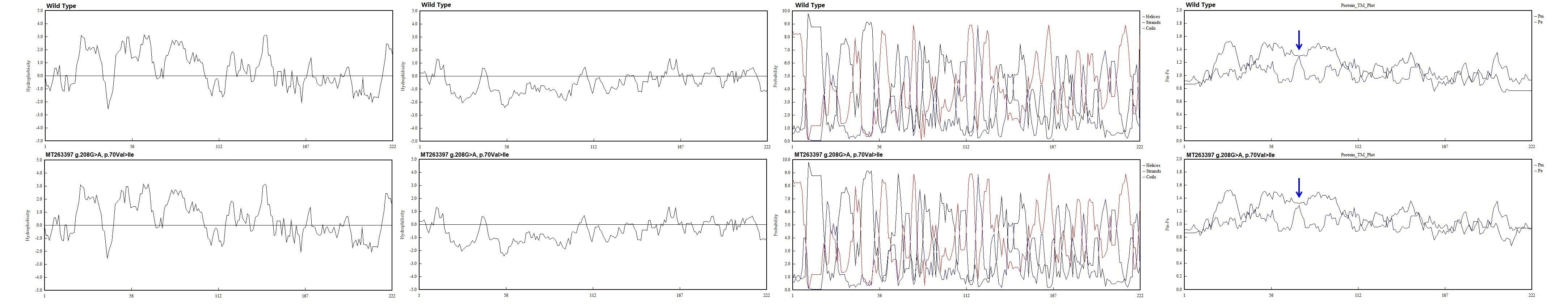

Supplement: Supplementary file 3 [file Image1.TIF]
